# Boosting Food Packaging Sustainability Through the Valorization of Agri-Food Waste and By-Products

**DOI:** 10.3390/polym17060735

**Published:** 2025-03-11

**Authors:** Angela Marotta, Angela Borriello, Muhammad Rehan Khan, Silvana Cavella, Veronica Ambrogi, Elena Torrieri

**Affiliations:** 1 Department of Chemical, Materials, and Industrial Production Engineering (INSTM Consortium—UdR Naples), University of Naples Federico II, P.le Tecchio 80, 80125 Naples, Italy; angela.marotta@unina.it (A.M.); veronica.ambrogi@unina.it (V.A.); 2 Department of Agricultural Sciences, University of Naples Federico II, Piazza Carlo di Borbone, 80055 Portici, Italy; silvana.cavella@unina.it; 3 Department of Agricultural and Food Sciences, Alma Mater Studiorum, University of Bologna, Piazza Goidanich, 47521 Cesena, Italy; muhammadrehan.khan2@unibo.it

**Keywords:** sustainable biopolymers, physiochemical properties, film properties

## Abstract

The environmental concerns associated with synthetic polymers have intensified the search for sustainable and biodegradable alternatives, particularly for food packaging applications. Natural biopolymers offer promising solutions due to their biodegradability, reduced environmental impact, and reliance on renewable resources. Among these, agri-food waste and by-products have gained significant attention as valuable feedstocks for polymer production, supporting a circular economy approach. This review critically examines the current status of biopolymers derived from plant, animal, and microbial sources, focusing on their physical and chemical properties and their application in food packaging. The findings underscore that the properties of plant- and animal-based biopolymers are heavily influenced by the source material and extraction techniques, with successful examples in biodegradable films, coatings, and composite materials. However, a critical gap remains in the characterization of microbial biopolymers, as research in this area predominantly focuses on optimizing production processes rather than evaluating their material properties. Despite this limitation, microbial biopolymers have demonstrated considerable potential in composite films and fillers. By addressing these gaps and evaluating the key factors that influence the success of biopolymer-based packaging, we contribute to the ongoing efforts to develop sustainable food packaging solutions and reduce the environmental impact of plastic waste.

## 1. Introduction

Nowadays, packaging preserves food quality and extends shelf life, which has a primary role in the food production chain and market [1]. Packaging not only has the role of protecting food from environmental agents, but it also plays a key role in the consumer’s perception of the food product [2]. In this frame, polymers have succeeded thanks to their low costs and highly versatile characteristics in processability and performance. Various polymeric systems, ranging from polyolefins to polyesters, have spread into the food packaging world for producing films for flexible packaging and/or coatings for active containers in food storage [3]. However, in recent years, growing attention to environmental issues has generated a strong concern about the use of petroleum-derived polymers typically used for food packaging applications, due not only to the scarcity of non-renewable resources but also to the lack of biodegradability [4,5].

In recent years, natural and biodegradable polymers have drawn much attention as a viable and environmentally acceptable substitute for traditional synthetic materials in various industries, including packaging applications [6]. Additionally, according to annual Food and Agriculture Organization (FAO) data on food loss and waste (FAO, 2023), agri-food waste and by-products have attracted huge attention as biomass renewable sources for the generation of polymeric materials [7].

**Figure 1:**
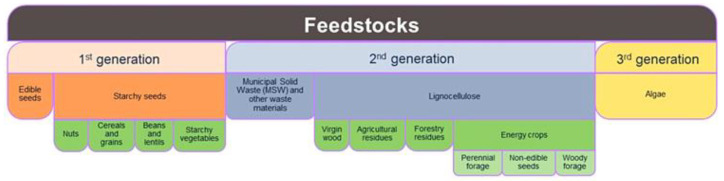
Biomass classification as feedstock.

Biomass to produce polymers to be used as structuring agents and fillers is mainly derived from animal, vegetable, and microbial wastes and by-products. Depending on their specific source, they are classified into first-, second-, and third-generation feedstock (Figure 1). The first-generation feedstocks are agricultural products, such as vegetable oils, corn, grain, and sugar. Although first-generation feedstocks are renewable, they compete with the food economy chain, encouraging deforestation and biodiversity loss. Second-generation feedstock can be partly addressed by producing monomers from non-food crops, industrial and urban waste, and agricultural and forest waste. This approach is expected to have significant positive consequences on reducing intensive cultivation and, thus, on costs. However, producing compounds from second-generation biomasses is still challenging due to biomass complexity requiring multiple pretreatment processes.

Third-generation biomass derives from microorganisms, such as algae and fungi, and it is a relatively novel strategy. The microbial sources have a high growth rate, limited land use, and do not require fertilizers or pesticides, being scarcely dependent on seasonal changes. Nevertheless, the technologies to produce biomolecules from third-generation feedstock are still at an early stage, and time is still needed to develop at a larger scale [8,9]. Despite all these critical issues, biomass as feedstock currently has a great potential as most of the challenges can be overcome, making it significant in the future. The potential application of second- and third-generation biomass in food packaging systems and its importance for the circular economy and environment has been extensively discussed in the recent scientific literature [10,11,12]. However, agri-food wastes and by-products present enormous challenges that need further and detailed evaluation to properly use them to produce packaging with the required function for food protection. The functional property of the biopolymer packaging depends on the chemical and physical properties of the biopolymer, which in turn depends on the specific source or the extraction/production process.

Understanding the physical and chemical properties of biopolymers derived from agri-food waste and by-products is crucial for tailoring the functional properties of biopolymer films for specific applications, such as packaging or coatings for food applications. Optimizing these properties, biopolymer films can offer sustainable and eco-friendly alternatives to conventional synthetic materials.

This review collects studies published in the last five years (2019–today) on biopolymers derived from agri-food wastes, with a specific focus on the physical and chemical properties of plant, animal, and microbial biopolymers and how the biomolecules have been successfully used to produce polymers and fillers for food packaging.

By comprehensively examining the properties of the biopolymer extracted from agri-food waste and by-products that play a crucial role in defining its success in producing food packaging materials, we contribute to the ongoing efforts to create a circular economy and reduce the environmental impact of waste disposal.

## 2. Methodology

**Figure 2:**
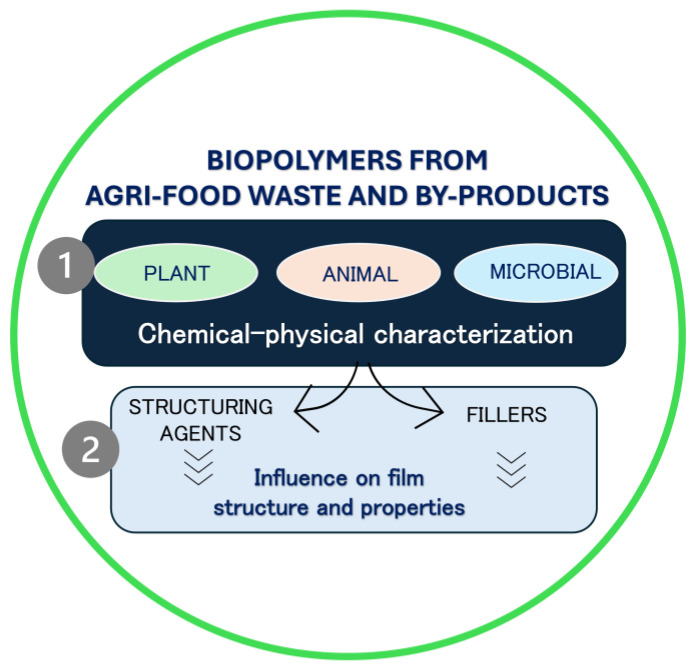
Schematic representation of review content.

Firstly, biopolymers recovered from agri-food waste and by-products were classified according to their origins as plant, animal, and microbial, and their chemical–physical properties were discussed. Secondly, we focused on the effects of biopolymers as structuring agents or fillers on the mechanical, structural, barrier, and functional properties of films or the rheological properties of film/coating-forming solutions (Figure 2).

**Figure 3:**
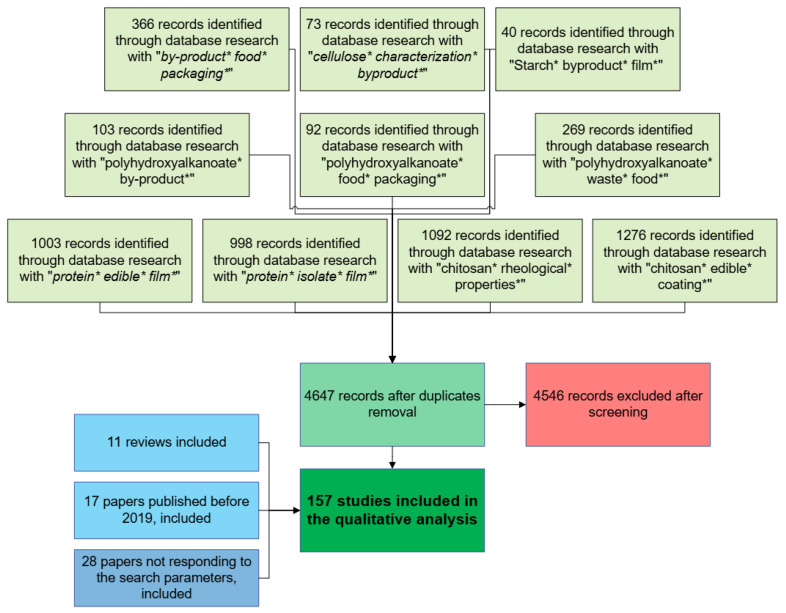
PRISMA diagram resuming the methodology followed in the preparation of this review.

The Preferred Reporting Item for Systematic Reviews and the Meta-Analytic (PRISMA) method was used. A library was generated by searching the Web of Science database, using the queries reported in Figure 3, and limiting the results to strictly article-type papers published in the last five years (2019–2023). The selection process followed a two-stage screening approach. In the first step, the titles and abstracts of the initial pool of articles were screened by two independent reviewers, removing those that did not meet the inclusion criteria. In the second step, a full-text review, the selected studies were further evaluated for eligibility, with any disagreements resolved through discussion or by consulting a third reviewer.

A total of 4647 articles resulted after duplicates were removed (i.e., research was performed combining all the queries with the OR Boolean operator). After title and abstract screening, 4115 studies were excluded. The remaining 532 full-text articles were assessed for eligibility, leading to the final inclusion of 101 studies. Additional references, including reviews and papers published before 2019 or not responding to the selected keywords, were included to ensure the completeness of the review. The PRISMA flow diagram (included in Figure 3) outlines the study selection process.

Inclusion criteria included (a) articles published in peer-reviewed journals between 2019 and 2023; (b) studies focused on biopolymers derived from agri-food waste and by-products; (c) investigations analyzing chemical–physical properties, structural characteristics, and applications in food packaging; and (d) research evaluating biopolymers as structuring agents or fillers for films and coatings. Exclusion criteria included (a) studies on biopolymers derived from non-food waste/by-products; (b) research lacking biopolymer characterization or not applicable to food packaging; (c) articles such as conference proceedings, book chapters, and patents; and (d) studies published in languages other than English.

For each study included, relevant data were extracted, capturing key information such as (i) the origin of the biopolymer (plant, animal, or microbial); (ii) the type of by-product used; (iii) physico-chemical properties, including molecular weight, chemical composition, solubility, and other physical characteristics specific to the polymer category (e.g., average diameter for cellulose, water-holding capacity for pectin, zeta potential for proteins, degree of deacetylation for chitosan, glass transition temperature for polyhydroxyalkanotes, etc.); (iv) the microbial strain used for producing biopolymers of microbial origin; and (v) applications in food packaging, including their roles as structuring or filler agents, as well as the structure and properties of films. A qualitative narrative synthesis was performed to identify trends, common findings, and gaps in the existing literature.

## 3. Biopolymers from Agri-Food Waste and By-Products for Food Packaging Applications

Recently, there has been increasing interest in using alternative renewable sources to fabricate biopolymers suitable for food packaging applications. Biopolymer packaging can extend the shelf life of food by controlling water vapor, oxygen, and carbon dioxide transmissions [13,14,15]. In addition, they can also be used as carriers of functional substances such as antioxidants and antimicrobials [16,17,18,19]. Using biopolymers from agricultural and food waste to replace fossil-based, non-degradable plastic materials represents a sustainable approach to overcoming environmental issues related to pollution, renewable resource depletion, and management of end-of-life packaging products. Depending on the sources and the synthesis, biopolymers can be divided into four classes: (1) polymers obtained from biomass, also known as agro-polymers, including polysaccharides (e.g., starches, cellulose, chitosan, alginate, pectin, carrageenan), animal proteins (e.g., gelatine, milk protein, muscle protein, egg white), vegetable proteins (e.g., soybean, wheat gluten, corn zein) and lipids (e.g., triglycerides, fatty acids, wax); (2) polymers obtained by microbial production, e.g., polyhydroxyalkanoates (PHA) such as poly(hydroxybutyrate) (PHB) and poly (3-hydroxybutyrate-co-valerate (PHBv), and others (e.g., pullulan, curdlan, xanthan, dextran, bacterial cellulose); (3) polymers chemically synthesized using monomers obtained from agro-resources (e.g., polylactic acid (PLA), polybutylene succinate (PBS), polybutylene adipate terephthalate (PBAT), polycaprolactone (PCL)); (4) polymers obtained by chemical synthesis from fossil resources (non-renewable resource) (e.g., polycaprolactones (PCLs), polyester amides (PEAs), aliphatic co-polyesters (e.g., PBSA), and aromatic co-polyesters (e.g., PBAT)) [20,21]. This review mainly focuses on the first two categories through the valorization of agri-food waste and by-products as sources for their production, which agrees with the principle of circular economy.

### 3.1. Physical and Chemical Properties of Plant-Origin Biopolymers

**Figure 4:**
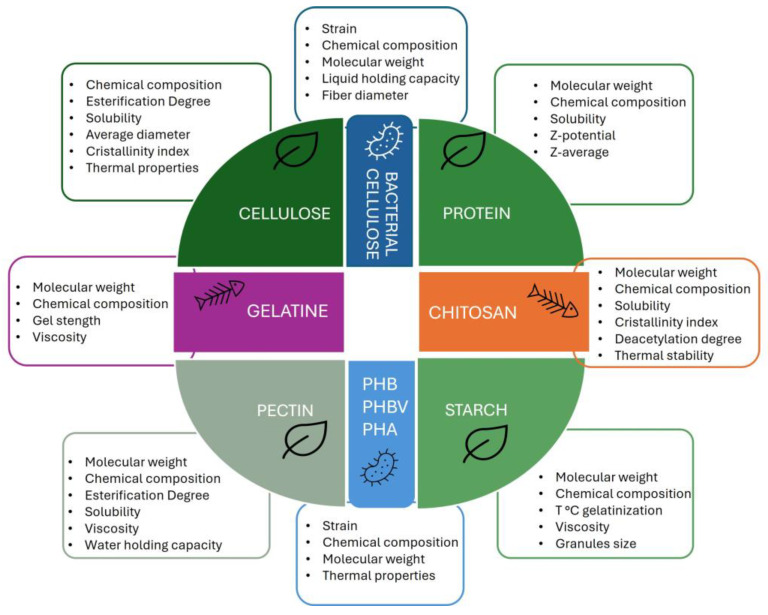
Physico-chemical properties of plant-, animal-, and microbial-origin biopolymers.

Natural biopolymers derived from plant sources or industrial waste and by-products show film-forming properties that exhibit various functional properties intricately linked to their molecular characteristics (molar mass, hydrophobicity, electrical charge). Understanding these relationships allows for the tailored use of biopolymers in various industrial applications, including food packaging. Table 1 describes the properties of plant-based biopolymers extracted from different agri-food waste and by-products. In detail, the molecular weight, chemical composition, solubility, and some physical properties that affect their applications in food packaging are reported, as depicted in Figure 4.

Cellulose is the most abundant polysaccharide in nature. It can be extracted from cotton and wood, the primary conventional sources, and from various high-fiber plants such as orange and banana peels. Cellulose can be further converted into different structures like micro- or nanocrystalline cellulose (MCC/NCC), cellulose microfibrils (CMFs), cellulose nanofibrils (CNFs), and nanowhiskers (CNWs) using various treatments and approaches [34]. Extracting cellulose from agri-food waste and by-products, particularly cellulose-rich ones, offers numerous ecological and economic advantages. For instance, Gabriel et al. explored teff straw, enset fiber, sugarcane bagasse, and coffee hull as alternative sources of cellulose using an eco-friendly and chlorine-free method [22]. The extraction and analysis of cellulose from various agri-food waste and by-products reveal significant differences in chemical composition. The findings indicate that cellulose extracted from coffee hulls stands out due to its higher contents of hemicellulose (8.8% *w*/*w* dry), lignin (7.9%), pectin matters (1%), and fatty/waxy matters (0.9%) contents. Shao et al. characterized the natural cellulose isolated directly from corncob, an agricultural waste after the grains were harvested [23]. The results showed that the particle size of the cellulose isolated showed a medium diameter of 83.34 μm. In addition, it had a higher degree of polymerization (581), a lower crystallinity (52.82%), and a lower cost than commercial MCC. MCC was prepared via acid (HCl) hydrolysis of cellulose isolated from date pits, showing a particle size ranging from 2.5 to 4 µm and a crystallinity index of 80.81% [24]. NCC and a lignin-enriched fraction were isolated in good yield from egagropili, better known as sea balls [27]. The determined fiber length was in the 1–2 mm range, whereas NCC chains exhibited a rod-like structure of 65–90 nm in diameter with smooth surfaces. It is well known that NCC is widely utilized as a reinforcement agent in polymer matrices for its peculiar properties, such as a high crystallinity and aspect ratio, large specific surface area, and abundance of surface hydroxyl groups able to form hydrogen bonds. Cactus fruit waste seeds (CWSs) contain an important amount of cellulose of about 27% and have been identified as a sustainable lignocellulosic source [25]. The authors reported that the CWSs contained 37 ± 3% lignin and 27 ± 2% cellulose, followed by a low ash content of 3.3 ± 0.4%. Moreover, they showed that CMFs from CWSs are appropriate for NCC production, exhibiting a needle-like shape with a diameter of 13 ± 1.4 nm and length of 419 ± 22.7 nm, giving rise to an aspect ratio of 30.7, with a zeta potential value of –30 mV and a charge content of sulfate groups of 287.8 mmol·kg^−1^. Typically, the aspect ratio plays a crucial role, primarily when CNCs are envisioned as nanofillers for polymer nanocomposite application, taking into account that an aspect ratio superior to 13 leads to the formation of an anisotropic phase within the polymer matrix, thus resulting in nanocomposite materials with improved properties. NCCs with a rod-like whisker shape, a crystallinity of 76.3%, and an average particle size of 309.4 nm were also produced from the brewery’s spent grain by-products using acid hydrolysis [35]. Another commonly underutilized agricultural waste is rice husk. A simple biorefining process was performed on rice husk to obtain fiber-like cellulose with a fiber width of 6 µm cellulose, tiny particles of hemicellulose and lignin with non-uniform shapes, and amorphous silica. Rice husk and straw from broken white rice have been successfully utilized to extract cellulosic nanocrystals to reinforce rice starch-based film [26]. The cellulosic nanocrystals from rice straw presented lower crystallinity (67%) and a more homogeneous morphology, with lower structural impurities, such as ashes and lignin, than those from rice husks. The latter showed a crystallinity index of 74% and large and agglomerated fibrillar structures.

Fruit and vegetable waste, such as peel, pomace, seeds, and pulp, are sources of several biopolymers, including pectin, starch, and mucilage. Many researchers have focused on developing novel coating materials and films by exploiting fruit and vegetable waste as a source of pectin [28,29]. Pectin shows non-toxicity, good film-forming properties, and good biodegradability, and it can improve the film’s extensibility due to its plasticizing role. The degree of methyl esterification (DM) of pectin refers to the percentage of carboxyl groups in the pectin molecule that are esterified with methyl groups. This parameter significantly affects the properties of pectin films as it influences their structure, mechanical properties, water solubility, and barrier properties. Depending on the DM of the carboxyl group, pectin can be classified as low methoxy pectin (<50% DM) and high methoxy pectin (>50% DM). The DM of pectin plays a critical role in determining the properties and performance of pectin films, making it a key parameter to consider when formulating pectin-based packaging materials for various applications. For example, the water solubility of pectin films decreases with an increase in the degree of methyl esterification. Pectin with a lower DM forms more water-soluble films, as the higher degree of methylation reduces the number of free carboxyl groups available for interaction with water molecules, resulting in decreased hydrophilicity and improved water resistance. In addition, pectin with a higher DM tends to produce films with increased strength and stiffness due to enhanced intermolecular interactions, including hydrogen bonding and electrostatic interactions, across pectin chains. Pectin can be extracted from citrus peel and pomace, watermelon and pomegranate peel, apple pulp and pomace, beet pulp, and pumpkin [20,36], but it can also be recovered from other sources such as cereals and soybeans. Frosi et al. characterized pectin extracted from conventional and emerging food waste sources, highlighting that one of the main factors determining pectin’s chemical and technological–functional properties is the source of the waste material [37]. An extensive review on the sustainable production of value-added products valorizing citrus peel waste was recently presented by Sharma et al., who proposed pectin recovery as a long-term strategy that may contribute to a circular economy and waste reduction [38]. Pectin from citrus peel differs from the one derived from other sources, showing higher viscosity, water solubility, and water-holding capacity. Most current research focuses on identifying new potential sources of pectin, such as grapefruit and red pomelo peels [28,29]. Khalil et al. developed novel active films entirely prepared with unused citrus peel waste. They compared the chemical characteristics of grapefruit peel pectin as a novel pectin source and commercial citrus pectin. The advantages of grapefruit peel pectin were due to the unique chemical structure and the high extraction yield. Compared with a commercial one, pectin from grapefruit showed superior characteristics regarding the degree of esterification (78.38%), methoxyl content (10.03%), and anhydrouronic acid content (88.10%), and also showed similar viscosity and water solubility. The extracted pectin yield was about 48% higher than that observed for orange waste and other fruit processing by-products. Grapefruit pectin was classified as high-methoxyl (>7%) and high-ester (>50%) pectin, indicating a good ability to form film [28]. By contrast, pectin isolated from pomelo peels can be categorized as low methoxyl pectin, disclaiming its potential application in film manufacture with optimal physical properties. Sood and Saini isolated pectin from red pomelo peel powder, containing both flavedo and albedo, to develop pure red pomelo peel pectin film and composite film together with casein and egg albumin. The pectin obtained had 84.13% purity, 50.75% degree of esterification, 7.10% methoxyl content, and 79.38% anhydrouronic acid content [29]. Film developed from pectin generally exhibits appropriate mechanical properties and an excellent barrier to oxygen but poor water resistance; this is the reason why pectin is combined with other biopolymers such as gelatine, chitosan, or sodium carboxymethyl cellulose in composite film-forming solution manufacture [39,40]. The physiochemical properties of pectin can also vary according to the cultivar or the ripeness stage. For example, Nguyen et al. investigated the properties of pectin extracted from the peel of three different mango cultivars at three different maturities [30]. Mango peel pectin was classified as high methoxyl pectin. Mango cultivars influenced the esterification degree, solubility, emulsion activity, and stability. Meanwhile, the water-holding capacity seemed to be mostly affected by the fruit’s maturation. Among polysaccharides, mucilage and starch derived from plant waste can also produce coating and film for food preservative purposes. The review by Beikzadeh et al. extensively described the structural fractions and food packaging application of mucilage extracted from seeds such as basil, psyllium, chia, and flaxseed [41]. Mucilage comprises polysaccharides, proteins, and other biopolymers contributing to its adhesive, film-forming, and thickening properties. Different seeds may yield mucilage with varying compositions and structural characteristics. Mucilage can form aqueous solutions with elastic properties comparable to those manufactured with synthetic polymers. Due to its adhesive, film-forming, and barrier properties, it has good potential for food packaging applications. A recent study proposed the mucilage obtained from Juazeiro fruit (*Ziziphus joazeiro* Mart.—*Rhamnaceae*) (yields 10–20%) as a novel renewable option to improve barrier properties of the coating, making it more effective in preserving the quality and freshness of the coated food products [42]. Starch can be extracted from multiple sources, such as cassava, potatoes, rice, wheat, corn, and peas, which are primarily used as food [43]. On the other hand, it can be recovered from several waste biomass sources such as industrial potato-washing slurries [31,32] and rice by-products [26]. The ability of starch to form films is influenced by factors such as its amylose and amylopectin content, molecular weight distribution, and granule size distribution. Higher amylose content in starch increases film strength and decreases water vapor permeability. Amylose molecules have a more linear structure, which promotes stronger intermolecular interactions and enhances film integrity. In contrast, higher amylopectin content contributes to greater flexibility and elasticity in starch films. The recovered starch from potato waste had an amylopectin/amylose ratio (2.3), gelatinization temperatures (59–71 °C), and a gelatinization enthalpy (12.5 J/g) similar to those observed for the commercial potato starch. However, its granules were more amorphous and produced lower viscous suspensions [31]. Due to its linear structure, starch with higher amounts of amylose is preferred for coating production. Benito-Gonzalez et al. analyzed the differences in the chemical composition of two starch-based fractions derived from rice waste: a less-purified fraction (rice flour) and a purified fraction (starch). The two starch-based fractions were highly comparable to starch, lipids, and ashes content and slightly differed in protein amount due to the applied method for the purification of starch, which reduced the number of proteins from 7% to 2%. Using less-purified starch-based material obtained from rice waste can yield more biocomposite-efficient materials from an economic and environmental perspective [26].

Food processing waste is also a source of proteins that can be used to develop bioplastics because of their cross-linking and film-forming abilities. Special attention should be paid to the waste of seed oil processing, which constitutes a potential renewable biological source of proteins. Proteins recovered from seed oil cakes of rapeseed, peanut, cottonseed, black cumin, sesame, and soybean were used to produce biodegradable films [44,45,46,47,48,49]. Among the most recent studies, Mirpoor et al. monitored the zeta potential and particle size of aqueous solutions of protein extracted from hemp seed oil cakes at different pH values to optimize the development of hemp protein-based films reinforced by microbial transglutaminase. The authors observed a protein-stable solution between pH 8.0 and 12.0, with protein diameters ranging from 400 to 500 nm, which increased as they approached the isoelectric point (pH = 6) until flocculation occurred. Hemp proteins could act as microbial transglutaminase substrates, such as proteins extracted from other seeds, achieving the highest degree of polymerization after two hours [33]. The functional properties of proteins depend on their solubility, which isolation techniques can influence. The emulsifying properties of hemp proteins are poorly investigated, primarily due to their low solubility. Dapčević-Hadnađev et al. compared the characteristics and emulsification ability of hemp protein isolated by two different isolation techniques: an alkaline extraction method and a micellization process. FTIR spectra and differential scanning calorimetry showed that the two types of proteins differ in their secondary structure. The alkaline conditions partially denatured the protein, which showed a lower solubility and gave solutions with a lower surface/interfacial activity than the protein obtained with the micellization method [50]. In conclusion, extracting polysaccharides and proteins from plant waste and by-products provides an opportunity to valorize waste materials, contributing to sustainability and resource efficiency. However, natural biopolymers exhibit various functional properties closely tied to their molecular characteristics, such as molar mass, hydrophobicity, and electrical charge. Understanding these relationships enables their tailored use in various industrial applications, notably in food packaging.

### 3.2. Physical and Chemical Properties of Animal-Origin Biopolymers

Biopolymers obtained from animal waste and by-products, such as collagen, gelatin, chitosan, keratin, and whey protein, have significant potential in food packaging due to their biodegradability, biocompatibility, and functional properties. Table 2 lists the molecular weight, chemical composition, solubility, and physical properties of animal-origin biopolymers, chitosan, and gelatin (Figure 4).

One of the most common animal-based biopolymers used in coating and film production is chitosan. Research on edible coatings based on chitosan has increased in recent years due to its advanced preservative properties, antimicrobial activity, and biodegradability. Chitosan (β-(1,4)-2-amino-2-deoxy-d-glucose) is a deacetylated form of chitin naturally contained in crustaceous and insect exoskeleton and in yeast, algae, and fungi. In acid media, chitosan is soluble and acquires antimicrobial properties since amines, upon obtaining protons and becoming positively charged, can interact with the negative surface of cell membranes, leading to bacteria inactivation. Its solubility is strictly related to the deacetylation degree and the molecular weight, which depends on the chitosan production process. The solubility increases with increasing deacetylation degree and lowering molecular weight [53]. Depending on the degree of deacetylation (DD), chitosan can be classified as low (55–70%), medium (70–85%), high (85–95%), or ultra-high (95–100%). Tavares et al. investigated the effect of DD on rheological properties and zeta potential of chitosan dispersions (3% *w*/*v* of chitosan in aqueous acetic acid solution 2% *v*/*v* at 20 °C) prepared by using chitosan with medium (83%), higher (94%), and ultra-higher (96%) DD. Higher viscosity, less sol–gel transition temperature, and stronger gel network structure were shown by chitosan with a higher degree of deacetylation. The zeta potential values increased from 13.00 to 20.65 mV with increasing DD, probably due to the increase in the protonated free amino fraction [54]. Moreover, depending on the extraction source, chitosan’s chemical–physical properties can be different. For instance, Luo et al. compared physico-chemical, rheological, and morphologic properties, including the solubility, molecular weight, and viscosity of chitosan extracted from four different insect shells with conventional chitosan extracted from shrimp shells. Chitosan obtained from insects showed higher solubility and thermal stability than shrimp chitosan. They also found that shrimp shell chitosan was suitable to prepare chitosan with high viscosity and molecular weight. In contrast, cicada slough chitosan could be used to obtain chitosan solution with low viscosity and molecular weight, while the other insects gave a medium viscosity chitosan [51]. The biological origin also affected morphologic properties, and a more compact structure was observed for cicada slough chitosan. Sometimes, the use of alternative sources can be sustainable and, at the same time, solve ethical problems, as in the case of gelatine. Gelatine, a water-soluble protein obtained from the partial hydrolysis of collagen, is widely used in coating and film production because of its film-forming properties, biodegradability, and edibility. Gelatine extracted from mammalian waste (e.g., bovine skin, beef bones, and pigskin), which is the main source of this biopolymer, is subject to criticism due to the risk of disease (e.g., bovine Spongiform Encephalopathy (BSE)) and for religious reasons (gelatine obtained from pork) [55]. Gelatine recovered from fish waste (skins, scales, and bones) is a promising alternative to mammalian gelatine but has low gelling ability due to low proline and hydroxyproline levels. Fish gelatin is often combined with natural polysaccharides, forming polyelectrolyte complexes with improved functional properties. Li et al. reported that gelatine extracted from the skin of a half-smooth tongue sole, a popular warm-water fish, had a high content of crude protein and low levels of ash, fat, and moisture, comparable to those observed for bovine commercial gelatine [52]. On the other hand, the obtained gelatine solution had lower strength values compared with bovine gelatine. The latter can be overcome by adding polymers that increase mechanical strength, such as chitosan [56,57], or agricultural waste extracts that improve rheological properties, increasing the intermolecular interactions [58]. Li et al. and Sekarina et al. reported that fish gelatine–chitosan blends form polyelectrolyte complexes due to electrostatic interactions between the chemically active groups of gelatine and chitosan, the negatively charged carboxyl group and the positively charged amino group, respectively [56,57]. Kaynarka et al. reported that adding grape pomace extract to fish gelatine recovered from Sparus aurata skin positively affected the kinetic affinity between the gelatine chains, increasing gel strength and gelation rate. Indeed, the functional properties of fish gelatine could be improved by the interactions between hydrophobic side chains of gelatine and phenolic aromatic rings, together with hydrogen bonds between the carboxyl groups of gelatine and the hydroxyl groups of phenolic compounds [58]. Other biopolymers from animal by-products include elastin, keratin, and whey protein. Elastin and keratin are generally recovered from fisheries, poultry, and red meat slaughter residues, and their properties strictly depend on the extraction method. Whey proteins are the primary by-products of milk manufacturing casein and cheese products. Elastin and keratin molecules, and also whey protein, spontaneously organize into networks stabilized by non-covalent interactions. These features, i.e., biodegradation, biocompatibility, and safety, make them ideal for application in coatings and films.

In conclusion, animal-derived biopolymers such as chitosan, gelatin, elastin, keratin, and whey protein possess significant potential for food packaging due to their biodegradability, biocompatibility, and diverse functional properties. These animal-based biopolymers underscore the potential of utilizing waste streams in sustainable packaging solutions, addressing both functional requirements and ethical considerations in biopolymer sourcing. However, the research field is of growing interest, yet still relatively young and developing compared to other sectors of biopolymer science, providing ample space for discoveries, innovations, and applications.

### 3.3. Physical and Chemical Properties of Microbial-Origin Biopolymers

Biopolymers of microbial origin derived from waste and by-products offer promising opportunities for sustainable food packaging applications. The physico-chemical characteristics of microbial-origin biopolymers obtained by different agri-food waste and by-products are summarized in Table 3.

Among the aforementioned polysaccharides, cellulose can also be produced by various bacterial species (such as *Komagataeibacter xylinus*) as an extracellular polymer of their metabolism known as bacterial cellulose. Bacterial cellulose builds up as bundles of ribbon-shaped nanofibrils with a higher degree of purity, crystallinity, and mechanical strength than plant cellulose. Citrus peels, commonly used as a source of pectin, can be important carbohydrate sources for bacterial cellulose production, thereby reducing production costs. Güzel and Akpınar compared the physico-chemical, structural, and thermal properties of bacterial cellulose produced from lemon, mandarin, orange, and grapefruit peels using *Komagataeibacter hansenii* GA2016. Although the crystalline nature of cellulose makes it insoluble in most organic solvents, it is characterized by its ability to retain liquids and swell. All the produced bacterial cellulose types showed high crystallinity, thermal stability, and liquid-holding capacity. Bacterial cellulose from lemon peels was found to have the highest water-holding capacity and thermal stability [59].

In contrast, the ones from grapefruit peels and orange peels had the highest crystallinity degree. The high production costs of bacterial cellulose, mainly due to culture media, can be decreased using alternative sources. For example, Sà et al. used cashew apple juice, yeast extract, and peptone as the culture medium for bacterial cellulose production [70].

Specific bacterial strains are also able to produce polymers with compositions and characteristics akin to conventional thermoplastics. These microbially derived biopolymers are polyhydroxyalkanoates (PHAs), linear polyesters constituted by 3-hydroxy fatty acids that accumulate as intracellular inclusions in the bodies of prokaryote microorganisms as a protection against oxidative stresses such as nutritional stress (limiting certain nutritional components) or environmental stress (unfavorable temperature, hypersalinity, UV radiation). The chemical composition of PHAs is a repetition of different polyhydroxyalkanoic acids, which can combine in the most varied way, resulting in biopolymers with completely different compositions and, specially, different mechanical and thermal properties. This biopolymer may be utilized in several applications across numerous sectors, especially food packaging and tissue engineering, thanks to its biodegradability, biocompatibility, thermoplastic behavior, hydrophobicity, and impermeability to gases and water. PHAs can be classified as short chain length (scl-PHA, three to five carbons in the acidic unit), medium chain length (mcl-PHA, six to four carbons), and long chain length (lcl-PHA, with fifteen or more carbons for each acidic unit). While the latter are scarcely produced, mcl-PHAs are the most industrially interesting as they are elastomeric-like with good adhesion properties [71]. Scl-PHAs, such as PHB and PHBV, are instead not very attractive for the industry due to their brittleness and poor mechanical properties. The use of agri-food waste and by-products can be inserted in PHA production practices using these wastes as a carbon source for substrates (i.e., products contained in water for bacterial growth), as well as a source of mixed microbial consortiums (MMCs), bringing both environmental and economic advantages [72,73,74]. In fact, besides the highly expensive controlled bacterial strains, the conventional carbon substrates, such as glucose, account for approximately 50% of the PHA’s production costs and can compete with the food chain [9]. Typically, agri-food waste and by-products intrinsically contain components, including starch, cellulose, and complex sugars, which are hardly converted by bacteria into the desired products or compounds with an inhibitory effect on bacterial growth. For example, the latter is the case of citrus peels containing limonene besides sugars. For this reason, waste and by-products often need to be pretreated to enhance their efficiency in PHA production. Corrado et al. proposed a cascade process to extract oil, phenols, lignin, and sugar hydrolysate from spent coffee grounds [75]. The obtained saccharification hydrolysates were used as the only carbon source to supplement *Escherichia coli*, which generated PHB, notwithstanding the presence of non-negligible amounts of furfural, 5-hydroxymethylfurfural, and proteins. Davaritouchaee et al., instead, found mild oxidation an efficient way to pretreat the orange peel particles and extract sugars to feed engineered *E. coli* JM109 [60]. In this way, the cell viability of the *E. coli* strain is increased by 90–100%, and a PHB with an M_w_ of ~1900 kDa and a crystallinity of 30–40% was obtained. Analogously, brewer’s spent grain must be physiochemically pretreated to obtain reducing sugars, which are a valuable substrate for PHA production in *Bacillus cereus*, *Cupriavidus necator*, and *Burkholderia cepacian* liquid cultures [76]. Similarly, Arias-Roblero et al. pretreated banana pulp by enzymatic maceration to convert the substrate into simple sugar molecules and increasing the accessibility of proteins and polysaccharides to *C. necator* [77]. Furthermore, when grape pomace extract was enzymatically treated and then used as a carbon source, the same bacteria proved to be very efficient in producing PHB with an M_w_ of 512 kDa [78]. Again, *C. necator* can produce PHAs from sweet potato pulp and rice wastes after saccharification [79,80,81]. Saccharification conditions of wheat waste biomass were instead optimized by Saratale et al. to improve the PHB production using *Ralstonia eutropha* [82]. Date fruit wastes contain up to 75% of reducing sugars produced by low-quality and spoiled or infested dates. The date syrup is, therefore, a valuable carbon source, as demonstrated by Madi et al. [83] and Chekroud et al. [84], for the accumulation of PHAs in high amounts by *C. necator* and *Bacillus paramycoides*. Date fruits can also be used as substrates for synthesizing PHBV by Haloferax mediterranei strains [63]. As reported by Gnaim et al. [64], *Cobetia amphilecti* successfully transforms [79,80,81] the acid hydrolysate of avocado seed waste into PHB with a high recovery yield, uniform molecular weight, and high purity. Molasses and olive oil, explored as substrates for PHA production by *C. necator*, could produce a polymer made of different methyl esters [85]. Volatile Fatty Acids (VFAs), generated by the acidogenic fermentation of organic wastes, are a further low-cost carbon source for producing different types of PHAs, depending on the composition and concentration of VFAs. They can be produced by anaerobic acidification of pressed sugar beet pulp [86], olive mill wastewater [87], or from the acidogenic fermentation of apple pomace and potato peel liquor, with the latter leading to the formation of PHBV with 9.6% of crystallinity using *C. necator* [66]. Similarly, an MMC with a 99% similarity with Thauera butanivorans was grown with alfalfa fermented hydrolysate (chosen among other fermented hydrolyzed from rice bran, animal epithelium, and soybean), producing PHBV [88]. The same copolymer is produced by batch fermentation using a mixture of fermentable sugars from corn straw as fermentation media for the growth of an MMC [89]. However, on the obtained PHAs, only spectroscopic studies have been performed in order to determine their chemical nature, but no physical properties have been tested [90]. To date, this remains a significant gap in the literature as most papers address the synthesis of PHAs from different kinds of by-products, such as cocoa bean shell [91], alkaline-pretreated cocoa pod husk hydrolysate [92], brewer’s spent grain [76,93,94], soybean hull [95], ragi husk [96], rapeseed meal [97], olive mill wastewater [98], spent coffee ground oil [99], palm oil mill effluent [100], sugarcane vinasse [101], sugar bagasse [102], sugar beet molasses [103], confectionery wastewater and rice parboiling water [104], rice wastewater [105], VFA-rich stream from potato peels [106], grape pomace [62,94], and plum waste juice [107]. These papers only focus on optimizing the production and extraction steps, but no information is given about the PHA’s physical and chemical properties.

Unfortunately, very few of the screened papers deal with PHB synthesis using agri-food waste and by-products as a substrate, together with the characterization of its molecular weight and thermal and mechanical properties. Among them, in a work by Maity et al. [67], PHB was produced by *Zobellellae tiwanensis* strain using fermented banana peels as a carbon source and without stress conditions; its mechanical properties were compared with those of a commercial PHB. The PHB produced by this agri-food waste, besides its biodegradable nature, is cytocompatible. Psaki et al. [68], instead, obtained PHB from *Paraburkholderia sacchari* using a solution from fruit-extracted free sugars as a feeding medium and found that the harvest time does not affect the thermal properties of PHB, while the bacterial strain and fermentation duration influence the physical properties and chemical structure of PHB. The effect of different purification processes on the physical characteristics of PHB produced by *C. necator* fed with grape pomace extracts was studied by Etxabide et al. [61]. They also evaluated the release of compounds from the purified PHB into food simulants, proving their applicability in food packaging. An mcl-PHA containing monomers with 6 to 14 carbons with saturated and unsaturated chains was obtained by Urbina et al. [65] using cider by-products as a carbon source for *Pseudomonas putida* strain. This biopolymer exhibits an elastomeric behavior, and the material is soft, elastic, and sticky at room temperature. The ability to produce spinnable PHA fibers produced from cassava peel residues through fermentation processes using *C. necator* was proven by Vega-Castro et al. [69] without further characterization of the polymer. Sequencing batch reactors (SBRs) can also convert different wastewaters into PHAs using different stains. Using SBRs, Traina et al. [108], Tamang et al. [109], and Guventurk et al. [110] produced PHAs from citrus processing wastewater, brewery wastewater, and pickle industry wastewater, respectively, using MMC. Morgan-Sagastume et al. [111] found that PHA production from the wastewater treatment of a potato starch factory on a pilot scale using a sequencing batch reactor proved the feasibility of the industrial use of waste and by-products for producing these microbial-derived polymers.

In conclusion, although diverse sources of agri-food waste can be used as carbon substrates for PHA production, underscoring their environmental and economic advantages, there remains a significant gap in the literature concerning the comprehensive characterization of PHAs synthesized from various waste and by-products, indicating a need for further research in this area to optimize their physical and chemical properties for practical applications.

## 4. Influence of Biopolymers from Agri-Food Waste and By-Products on Film Structure and Properties

In the field of coatings and films for food packaging applications, agri-food waste and by-products have been utilized not only as a source of biopolymers such as chitosan, gelatin, pectin, and cellulose [20] but also as potential sources of compounds to be used as filler.

**Figure 5:**
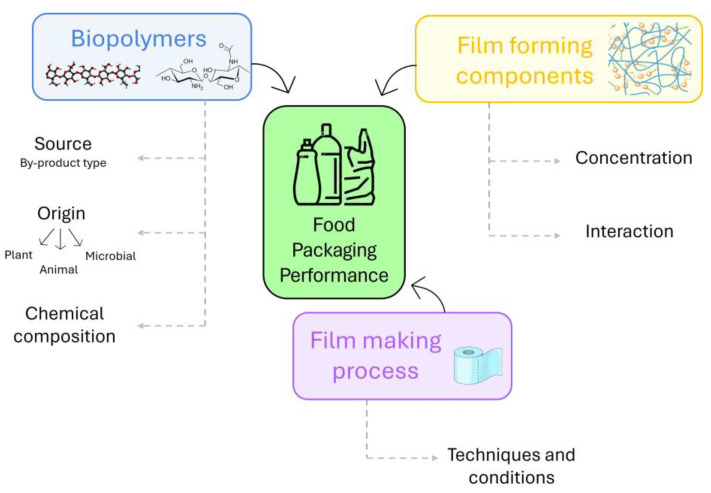
Key factors affecting agri-food waste and by-product food packaging performance.

The following section discusses how biopolymers derived from agri-food waste and by-products were recently effectively utilized as structuring compounds, alone or in combination with other polymers, and as fillers of films with specific physical properties. Furthermore, the detailed relationships among biopolymer interactions, the structure of films/coatings, and their properties have been discussed in order to select a biomaterial for future research in this area. A schematic diagram portraying the significant factors that influence food packaging from agri-food waste and by-products is depicted in Figure 5.

### 4.1. Structuring Biopolymers

Biopolymers derived from agri-food waste, i.e., proteins and polysaccharides, are commonly utilized to develop composite biodegradable films and coatings with appropriate physico-chemical properties. Generally, polysaccharides provide excellent mechanical properties, while proteins provide good gas barrier properties, and using them in combination improves the performance of films and coatings [112]. The tensile properties of the composite films, prepared with biopolymers derived from agri-food waste and by-products, significantly improved due to a more compact structure as a result of the intermolecular interactions increasing among polymeric chains and reduced molecular mobility [113]. However, tensile properties are significantly affected by intra- and intermolecular interactions, the concentration of the film compounds and their nature, and the preparation procedures [112]. For instance, composite films of casein and egg albumin and pectin (obtained from red pomelo) displayed significantly higher tensile strength with increasing concentrations of pectin (from 25 to 50%); on the other hand, elongation at break (%) decreased significantly (from 18 to 4%) due to improved compactness of the film structure and the restricted chain mobility. Similarly, Khalil et al. [28] observed almost two times higher tensile strength for high methoxyl pectin-based films extracted from grapefruit peel due to increased interactions between bioactive compounds, such as polyphenols and pectin molecules as compared to films made from commercial citrus pectin. On the other hand, Li et al. [56] reported a significant reduction (9–21%) in the tensile strength of the films (chitosan and gelatin) upon the incorporation of orange peel extract oil (0.25–1% *v*/*v*) due to structural discontinuity induced by weak intermolecular interactions between oil and polymeric components.

Generally, the chemical, crystalline, and microstructural properties of biopolymeric films are influenced by film-forming components, ultimately affecting the films’ mechanical, barrier, and biodegradation properties [114]. The correlation among structural properties, surface properties, and barrier properties of films is of primary importance in tailoring the film properties for specific applications. Understanding these correlations enables the design of packaging materials with optimized performance, meeting the required food preservation and safety standards. For instance, FT-IR analysis is performed to understand the interactions of functional groups of interest across polymeric chains, and any variation in the FT-IR spectra can highlight changes in the functional properties of the films; thus, as an example, the correlation between the change in spectral position and intensity with physical properties is critical to select an appropriate biopolymer from vegetable waste and by-products for inclusion in the packaging material formulation to improve water resistance. The plant-derived polysaccharide-based films display stretching vibration of bound and free hydroxyl groups of carboxylic acid in the 3200–3600 cm^−1^ region. However, this can also be correlated with stretching vibrations of O-H groups due to intra- and intermolecular H-bonding between monomers of the polymeric chains [29,30]. The stretching of the C=O group can be observed at 1700–1750 cm^−1^; however, the intensity of this peak can vary depending on the degree of methoxylation or esterification due to interaction with other film-forming components [30].

Furthermore, for composite egg albumin, casein, and pectin films, changes in intensity and position of the peaks were observed for amide-A and amide-I regions due to higher interactions between polymer molecules, which resulted in higher tensile strength and lower permeability [28,115]. Another important phenomenon reported in the literature was the shifting of O-H spectra from 3200 cm^−1^ to 3400 cm^−1^ for soy protein-based hydrogels when soybean polysaccharides (from 0 to 5%) were added, which led to strong hydrogen bonding. Furthermore, the electrostatic interactions between -NH groups in soy protein and -COO^−^ in the polysaccharide led to the disappearance of C-N and N-H bending vibrations at 1540 cm^−1^, which can be correlated with an increased contact angle of the gels at the highest polysaccharide concentration [116]. XRD analysis allows us to analyze the degree of crystallinity of the polymeric matrix. Generally, biopolymeric films from agri-food waste and by-products display characteristic peaks between 2θ = 11 and 20°, indicating the amorphous to semi-crystalline structure of the polymeric materials. Thus, increasing the amorphous phase allows more water molecules to interact with the polar groups on the polymer surface. For instance, when grapefruit peel was blended with fish gelatin and PVA, the peak of 2θ changed from 19.34 to 19.30° due to structural repositioning caused by the interaction between grapefruit peel, gelatin, and PVA, highlighting a decreasing degree of film crystallinity [117]. Similarly, Su et al. [118] reported a significant reduction in the crystallinity of composite soy protein isolate/carboxy methyl cellulose films due to the incorporation of carboxymethyl cellulose that modifies soy protein isolate’s crystalline structure. Ghosh and Katiyar observed improvement in the crystallinity of the potato starch coating after the addition of guar gum at 2θ = 16 and 22.7° due to interfacial interactions between both the components at an intermolecular level, which reinforced the surface structure and is also outlined by the reduced peak intensity at -OH, -CH, and –CO stretching vibrations [119].

The microstructural analysis is robust in investigating the impact of combining different biopolymers derived from fruit and vegetable waste and by-products on the structure and performance of the films. The goal is to achieve a more compact and homogeneous film without phase separation, detected by the absence of micro holes and cracks. Sood and Saini found that the film matrix became more compact when red pomelo peel pectin was incorporated into composite films of pectin, casein, and egg albumin, with a decrease in water vapor permeability, elongation at break, solubility of the films, and improved tensile strength [29].

The barrier properties of films are crucial in food packaging to prevent the transfer of water vapor and oxygen into the food product, thereby preserving its quality and extending shelf life. The composition of the films significantly influences these properties, particularly when combining proteins and polysaccharides. Generally, films made from plant-based proteins (e.g., soy protein and wheat gluten) have high water vapor permeability due to protein chains’ voluminous and flexible nature, which creates larger free volumes within the film matrix, easing water molecules diffusion. While plant-based proteins provide a basis for film formation, their high-water vapor permeability can be significantly reduced by incorporating tightly packed polysaccharides such as carboxymethyl cellulose, chitosan, gum Arabic, and pectin. These polysaccharides enhance the interactions between polymer chains, creating a dense network that lowers water vapor permeability (WVP) [120]. Similar trends were observed by Li et al. [121], which reported a significant reduction (from 167 to 139× 10^−11^ g m/s/Pa) in WVP of a film based on the protein isolate obtained from peanuts upon the incorporation of gum Arabic. Their results agreed with the more compact structure of the film, as revealed by SEM images.

Film and coating surface properties are also crucial for evaluating their performance in food packaging applications. One important surface property is the contact angle, which measures the wettability and water resistance of the film or coating. Biopolymer films derived from animal- and plant-based sources exhibit distinct surface properties influenced by various factors such as their origin, concentration, extraction methods, chemical composition, and intermolecular interactions [122]. The enhancement of surface hydrophobicity (from 48 to 93°) observed in xerogels derived from soy protein upon the incorporation of soybean polysaccharides (from 0 to 5%) is a significant finding that underscores the impact of composition on the surface properties of biopolymer films. It is attributed to stronger hydrogen bonding interactions between the soy protein and polysaccharides. FT-IR spectroscopy reveals shifts in the amide-II and amide-III bands of the soy protein component upon adding polysaccharides. These shifts indicate structural changes and interactions within the biopolymer matrix, suggesting closer association and possibly cross-linking between protein and polysaccharide molecules. The resulting compact structure of the film, with reduced porosity and a denser network, slows the diffusion of water molecules through the xerogels [116].

Adding hydrophilic polymers like guar gum to starch-based coatings incorporated with nano chitosan decreases surface hydrophobicity due to increased interactions with water molecules via hydrogen bonding. This interaction alters the surface energy and reduces the contact angle, indicating a more wettable surface. The observed changes in FT-IR spectra, particularly the reduction in C=C (<1645 cm^−1^) bond intensity, further confirm the modification of surface chemistry [119]. On the other hand, polysaccharides of animal origin, i.e., chitosan, can significantly impact the surface hydrophobicity of the films and coatings. For instance, chitosan coating increases the surface hydrophobicity of the starch films (up to 30 times) due to hydrophobic acetyl groups, which can also hinder the transport of water molecules through the film or coating. However, the hydrophobic property of chitosan is always dependent upon incomplete deacetylation [123]. It is not always valid for protein-based biopolymers, irrespective of their source, due to the presence of hydrophilic groups [124]. Contrarily, the contact angle of the films/coatings increased after incorporating hydrophilic biopolymers, i.e., gelatin and soy protein. This increase is attributed to the exposure of hydrophobic groups on the surface of the biopolymer matrix, caused by strong interactions between gelatin/soy protein molecules and potentially by the rearrangement or exposure of hydrophobic regions within the film structure. This hypothesis was confirmed by the disappearance of characteristic peaks, such as those observed at 1332, 1198, and 1028 cm⁻¹, suggesting structural modifications or interactions of the gelatin or soy protein molecules [125,126].

**Figure 6:**
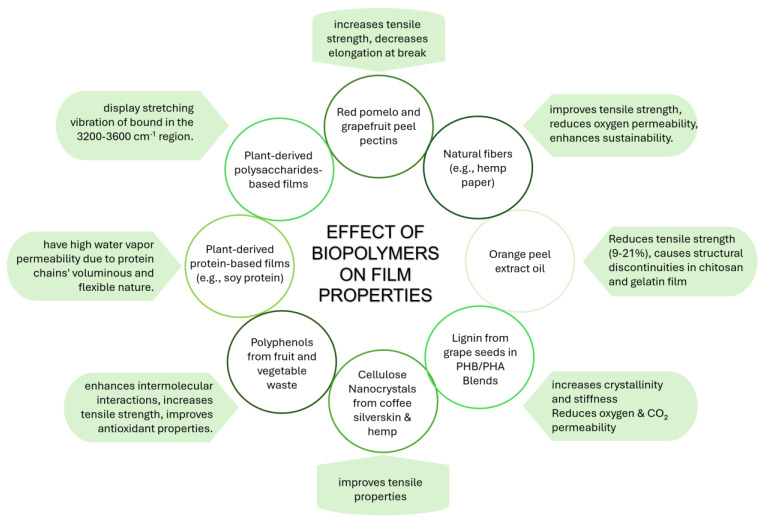
Effect of biopolymers from agri-food waste and by-products on film properties.

The most used PHA, especially in food packaging applications, is PHB, despite its poor processability and thermal stability. The main way to overcome this problem is to blend PHB with poly(3-hydroxybutyrate-co-3-hydroxyvalerate) (PHBV), a copolymer of PHB and 3-hydroxyvalerate acting as a plasticizer. PBHV has been used in the manufacturing of electrospun mats for food packaging applications [127,128,129] or blended with ferulic acid and p-coumaric acid to produce films with antibacterial activity [130]. When loaded with tannins, PHBV can also be used as multifunctional active food packaging, suitable for colorimetrically detecting food spoilage [131]. PHBV produced by *Wickerhamomyces anomalous* supplemented with sugarcane molasses and palm oil, loaded with biogenic silica nanoparticles, showed not only an improvement in thermal, mechanical, heat resistance, and barrier properties due to the presence of the filler but also excellent biocompatibility, biodegradability, and antimicrobial properties [132]. An interesting approach for the production of P(3HB)/mcl-PHA blends was proposed by Rebocho et al., which directly produced the blend (composed of nearly equal contents of each biopolymer) by a co-culture of *C. necator* and *P. citronellolis* using apple pulp waste as the sole carbon source [133]. The processing of this blend led to flexible and elastic films with mechanical properties dominated by the contribution of the mcl-PHA phase and permeability to oxygen and carbon dioxide comparable to those of P(3HB). PHB is generally blended with different bioderived and biodegradable polymers such as soybean protein [134], PBAT [135,136], PLA [137,138,139], PBSA [140,141], and pectin [142,143]. Also, PHAs with different structures can be blended to obtain a film with improved thermal, mechanical, and gas barrier properties [144]. Meléndez-Rodríguez and coauthors [145] synthesized a terpolymer, P(3HB-co-3HV-co-3HHx) from an MMC fed with fruit pulp biowaste and used it as a plasticizer for a commercial PHB. The resulting film also showed reduced oxygen, water, and limonene vapor permeability. Peanut oil was used by Pérez Arauz et al. [146] to stress *C. necator* during the fermentation step of PHA production, successively used for the production of films with good barrier properties but reduced biodegradability. *P. citronellolis* is successfully produced from apple pulp waste, mcl-PHA, used to produce films by solvent casting [147]. However, they exhibit poorer oxygen and water barrier properties than commercial PHB. The good properties of PHA were further exploited using this biopolymer in the production of layered composites with natural fibers, such as hemp paper [148] or bacterial nanocellulose [149]. Fully PHBV multilayer films, composed of a layer of pristine PHBV, electrospun eugenol-loaded PHBV, and food-contact PHBV, were obtained by Figueroa-Lopez et al., ensuring a good interlay adhesion [150]. These films possess antibacterial properties, hydrophobicity, improved barrier properties against water and aroma vapors, and were proven to regulate and reduce the growth of food-borne bacteria for up to 15 days thanks to the release control of eugenol. Active films were also obtained by thermal annealing of electrospun PHB loaded with different essential oils [151], possibly loaded in mesoporous silica nanoparticles [152]. Pereira et al. [61] showed that from apple peels, it is possible to obtain mcl-PHAs with high M_w_ (1.34 × 10^5^ Da) from which highly flexible films, exploitable as food packaging, can be produced. The PHBV obtained by feeding bacteria with date fruits [63], loaded with silver-doped ZnO nanoparticles, inhibits *Staphylococcus aureus* and *E. coli* growth [153]. A PHB/PHV copolymer was used as a substrate for the deposition of β-carotene-loaded electrospun fibers of soy protein isolate/polyvinyl alcohol with potential application as active food packaging (due to the tunable release of β-carotene by annealing processes) [154]. Further, Moreno et al. [155] deposed zein fibers loaded with phenolic-enriched extracts from orange pulp, seed, and skin, producing films with analogous characteristics of active compound release. As Plouzeau et al. [156] proved, PHBH can be recycled and used for food packaging production. In fact, after several reprocessing cycles, PHBH loses its mechanical properties, but moisture resistance is unaltered, and the gas barrier properties are enhanced. The safety concerns that may arise from the use of waste-derived PHA in food contact applications are overcome by the study conducted by Astolfi et al. [157], who analyzed the potential migration of contaminants transferred from the bio-waste to PHA to potentially packed foods. This study shows that the migration of heavy metals is always below the limits imposed by the European Commission for materials intended to come in contact with refrigerated foods. The main effects of using different resources and fillers on the final properties of the films are summarized in Figure 6.

### 4.2. Filler Biopolymers

To improve the mechanical, barrier, and rheological properties of films/coatings, filler biopolymers (extracted from agri-food by-products) can be added. These substances are non-toxic and non-reactive. Depending on the type of filler, the concentration utilized, and the interactions with other compounds, the properties of biopolymeric films and coatings can be modified. Clays, cellulose derivatives, and chitosan are some of the filler biopolymers most frequently utilized [158]. Filler biopolymers can positively affect the films’ mechanical properties due to the cross-linking effect. For instance, the addition of cellulose nanocrystals improved the tensile strength of the starch films (i.e., 10 folds) due to its influence on the gelatinization of starch during processing and its subsequent recrystallization. Moreover, the effect is also dependent upon the source of nanocrystals (i.e., rice husk or rice straw) [26]. Similarly, cellulose films incorporated with cellulose nanocrystals and lignin showed significant improvement in strength and modulus due to two reasons: (1) lignin formed hydrogen bonds with the matrix, increasing stiffness, and (2) cellulose nanocrystals formed a percolating network within the film matrix due to strong adhesions at the nanocrystal–matrix interface, promoting transfer of stress from the matrix towards nanocrystals [70]. However, nanocrystals can even further improve the tensile properties of the films if added during cellulose biosynthesis, as shown by Butchosa et al. [159] for chitin nanocrystals. Lignin isolated from grape seeds was, instead, used by Vostrejs et al. [160] as filler in PHB/PHA blends as a radical scavenging additive, contextually increasing crystallinity and stiffness of the film and lowering their oxygen and carbon dioxide permeability. Although adding lignin preserves these films’ biodegradability, it has been proven that white mustard germination could be accelerated by the biomass left over from the film’s composting process. Further, a blend of PHB/mcl-PHA, loaded with phloretin (a flavonoid found in fruits and vegetables), with antimicrobial, antioxidant, and food preservative properties, was produced by Mirpoor et al. [161]. Other sources of cellulose nanocrystals include coffee silverskin and hemp, which were used as nanofillers to improve the tensile properties based on the cross-linking effect [162,163,164]. However, the use of silverskin itself as a filler has been negatively associated with an improvement in mechanical properties [165].

Adding fillers can result in structural property changes in films and coatings. For instance, it can improve the intensity of specific functional groups, i.e., CH_2_ symmetric bending and C-O-C asymmetric bending, due to the incorporation of cellulose nanocrystals into the cellulose-based films. It can also shift C=O stretching vibrations to a higher wavenumber as a consequence of the inclusion of the lignin filler, which occurs due to the hydrogen bonding between the filler and biopolymer, leading to higher surface hydrophobicity in the films [70]. Li et al. [56] reported a significant reduction in hydrophilic groups upon the incorporation of orange peel extract oil (from 0 to 1% *v*/*v*) in fish skin gelatin/chitosan films, which was confirmed by shifting amide-A spectra to a higher wavenumber, and it was found to be correlated with a higher contact angle compared to control film. Furthermore, an improvement in -OH stretching vibrations can be observed upon intra- and intermolecular interactions between filler material and base biopolymers. For instance, lignocellulosic hemp particles can shift the OH spectra of PVA films to a higher wavenumber (from 3289 to 3337 cm^−1^) due to hydrogen bonding, as the OH groups of lignin can form hydrogen bonds with the PVA matrix. Also, biopolymers from microbial origin can be used as filler. The efficiency of PHA in encapsulating cinnamaldehyde was proven by Yang et al. [166], who assessed their ability to prevent strawberry spoilage. Further, Corrado et al. [167] successfully encapsulated plant-derived essential oils into PHA-based nanoparticles, potentially available to produce active food packaging.

## 5. Conclusions and Future Trends

The valorization of agri-food waste and by-products to produce packaging materials is gaining significant attention due to its potential to address environmental concerns, reduce waste, and create sustainable alternatives to conventional packaging materials. The review discusses the potential of transforming waste into by-products as they can be a source of biopolymers to produce films and coatings. The work emphasizes the need to thoroughly investigate the chemical–physical characteristics of plant, animal, and microbial biopolymers and how these can change depending on the source material and extraction processes. Furthermore, there is a significant gap in the literature regarding microbial biopolymers’ physical and chemical properties, a topic that remains underexplored. Recent research has been focusing on optimizing production processes to overcome these gaps, but further investigation is necessary to fully understand how these materials can be used effectively in packaging applications. Results also highlight the importance of a comprehensive understanding of the biomaterial interactions when used as structuring agents or fillers and the corresponding effect on different physico-chemical properties of films to select an appropriate biomaterial for further investigation in this field. Despite the advancements made in this area, several challenges persist, particularly in achieving uniformity in film properties and scaling up production processes. One of the primary obstacles in scaling up the production of food packaging from agri-food waste and by-products is the inherent variability in the composition and quality of the waste materials. This variability makes it challenging to standardize production processes on a larger scale and can affect the consistency and performance of the final product. Furthermore, the transformation processes required to convert food waste into packaging materials can be complex and costly. If these processes are not optimized, the costs involved in production may approach or even exceed those of conventional materials, undermining the economic viability of the technology. Additionally, the current infrastructure for the collection and processing of food waste is often inadequate, requiring significant investments in new facilities and industrial processes to enable large-scale production. Looking ahead, the author believes that overcoming these challenges will require a multi-faceted approach. Future research should focus on optimizing the extraction and processing techniques to ensure cost-effective and scalable production of biopolymer-based films. In conclusion, while there are significant hurdles to overcome, the potential for food packaging made from agri-food waste and by-products remains immense. With continued research and development, it is possible to enhance the practicality and scalability of biopolymer-based films, paving the way for a more sustainable and circular approach to food packaging.

## Figures and Tables

**Table 1:** Physico-chemical properties of plant-origin biopolymers obtained from different agri-food waste and by-products.

Biopolymer	By-Product	Molecular Weight	Chemical Composition	Solubility	Physical Properties	Ref.
cellulose	teff straw	-	Cellulose (89.9%); Hemicellulose (4.6%); Klason lignin (3.1%); Pectic matters (0.3%); Fatty and waxy matters (0.4%); Aqueous extractives (2.2%); Ash (0.6%)	Insoluble in water, acetone, anhydrous ethanol, and toluene. Soluble in cuprammonium hydroxide ‘Cuam’ solution.	Average diameter (μm) 6.39 ± 2.24	[22]
	enset fiber	-	Cellulose (95.7%); Hemicellulose (2.3%); Klason lignin (0.7%); Pectic matters (0.3%); Fatty and waxy matters (0.2%); Aqueous extractives (0.3%); Ash (0.7%)	Insoluble in water, acetone, anhydrous ethanol, and toluene. Soluble in cuprammonium hydroxide ‘Cuam’ solution.	Average diameter (μm) 13.37 ± 2.45	[22]
	sugarcane bagasse	-	Cellulose (90.8%); Hemicellulose (4.0%); Klason lignin (2.8%); Pectic matters (0.7%); Fatty and waxy matters (0.5%); Aqueous extractives (0.6%); Ash (0.7%)	Insoluble in water, acetone, anhydrous ethanol, and toluene. Soluble in cuprammonium hydroxide ‘Cuam’ solution.	Average diameter (μm) 19.19 ± 5.18	[22]
	coffee hull	-	Cellulose (79.9%); Hemicellulose (8.8%); Klason lignin (7.9%); Pectic matters (1.0%); Fatty and waxy matters (0.9%); Aqueous extractives (0.7%); Ash (1.0%)	Insoluble in water, acetone, anhydrous ethanol, and toluene. Soluble in cuprammonium hydroxide ‘Cuam’ solution.	Average diameter (μm) 24.06 ± 7.37	[22]
	corncob	-	Degree of polymerization (581)	-	Average diameter (μm) 83.34 Average pore size (nm) 17.95 Crystallinity index (%) 52.82	[23]
	date pits	-	-	-	Particle size (µm) 2.5–4 Crystallinity index (%) 80.81 Thermal degradation (°C) 340–345	[24]
	cactus fruit waste seeds	-	Cellulose (27%); Lignin (37%); No hemicellulose	-	Needle-like shape Diameter (nm) 13 ± 1.4 Length (nm) 419 ± 22.7 Crystallinity index (%) 86 Zeta (-mV) −30.0 L/D 30.7 T_onset_ (°C) 273 T_max_ (°C) 340 the 5% CNC suspension behaves as a gel-like structure	[25]
	rice husks	-	Arabinose trace; Galactose trace; Glucose (%) 89.9 ± 14.5; Xylose (%) 10.1 ± 1.7; resistant lignin remained linked to the residual ash fraction	-	Thermal degradation (°C) 330 Crystallinity index (%) 74	[26]
	rice straw	-	Arabinose trace; Galactose trace; Glucose (%) 83.3 ± 4.4; Xylose (%) 16.7 ± 1.2	-	Thermal degradation (°C) 330 Crystallinity index (%) 67	[26]
	egagropili	-	-	-	rod-like structure of 65–90 nm	[27]
pectin	grapefruit peel		Ash (9.0%); Anhydrouronic acid content (88.10%); Degree of esterification (78.38%); Eq. wt (g/mol) 1650.0; Moisture content (6.82%); Methoxyl content (10.03%); Yield (%) 48.35	water solubility 80.56%. below pH 4, water solubility is poor as it is high-methoxyl pectin, where an increase in the number of negatively charged free hydroxyl increases the repulsive forces, resulting in weaker gels	Viscosity (mPas) 20.27 Water-holding capacity (g H_2_O/1 g pectin)10.76	[28]
	red pomelo peel	-	84.13% purity; 50.75% degree of esterification; 7.10% methoxyl content and 79.38% anhydrouronic acid	Soluble in distilled water (800 rpm for 1 h)	-	[29]
	mango peel	397–578 kDa	49.6–55.8% degree of esterification	Nanopure water solubility 77.4–87.4%	Intrinsic Viscosity 44.5–73.7 (mL/g) Emulsion activity 11.8–34.2% Emulsion stability 28.5–94% Water-holding capacity (g H_2_O/1 g pectin) 9.5–14.9	[30]
starch	potato washing slurries	amylopectin 350 kDa amylose 50 kDa	Amylopectin/amylose ratio 2.3	gelatinized in distilled water at 95 °C for 30 min with continuous stirring at 300 rpm	Viscosity 612 mPa·s (90 °C). Spherical and ovals granules (15.4–62.7 μm)	[31,32]
	rice flour		Protein 2.0 ± 0.2; Lipids 1.7 ± 0.0; Ashes 1.8 ± 0.2; Xylan 18%; Arabinose/xylose ratio of 1:5	-	-	[26]
protein	hemp seed oilcake	HP SDS-PAGE profile showed three major bands with molecular masses of ~35 kDa, ~19 kDa, and ~16 kDa, identified as the primary proteins occurring in the hemp protein concentrate.	The main protein content of hemp seeds consists of albumin and edestin, polypeptides with high amounts of arginine, glutamic acid, as well as of sulfur-containing amino acids, which makes their amino acid profiles comparable with those of soybean, egg, and meat proteins	soluble in distilled water (0.1 mg/mL) under constant stirring at pH 12.0	Zeta potential of aqueous solutions of hemp protein concentrate (72% HPs) is −31 mV (pH 8.0), −24 mV (pH12.0), −17 mV (pH 6.0), −4 mV (pH 5.0). Z-average size is 400–500 nm (pH12.0–8.0), >1000 nm (pH < 7.0)	[33]

**Table 2:** Physico-chemical properties of animal-origin biopolymers obtained from different agri-food waste and by-products.

Biopolymer	By-Product	Molecular Weight	Chemical Composition	Solubility	Physical Properties	Ref.
chitosan	Cicada slough	3.779 × 10^4^ Da	Ash 0.03% Moisture content 0.18%	1% aqueous acetic acid at 30 °C 99.3%	Crystallinity index 64.8% Deacetylation degree 84.1% Thermal stability 48% T_gel_ 35 °C	[51]
	Silkworm chrysalis	4.090 × 10^4^ Da	Ash 0.05 Moisture content 0.07%	1% aqueous acetic acid at 30 °C 98.7%	Crystallinity index 32.9% Deacetylation degree 85.5% Thermal stability 45%	[51]
	Mealworm	3.975 × 10^4^ Da	Ash 0.50% Moisture content 0.19%	1% aqueous acetic acid at 30 °C 97.4%	Crystallinity index 51.9% Deacetylation degree 85.9% Thermal stability 39% T_gel_ 33 °C	[51]
	Grasshopper	3.989 × 10^4^ Da	Ash 0.89% Moisture content 1.8%	1% aqueous acetic acid at 30 °C 94.3%	Crystallinity index 50.1% Deacetylation degree 89.7% Thermal stability 34% T_gel_ 53 °C	[51]
	Shrimp shell	1.620 × 10^5^ Da	Ash 0.90% Moisture content 2.7%	1% aqueous acetic acid at 30 °C 91.5%	Crystallinity index 49.1% Deacetylation degree 91.2% Thermal stability 27%	[51]
gelatine	Half-Smooth Tongue Sole skin	53–220 kDa	Protein (g/100 g) 89.76 Fat (g/100 g) 0.81 Moisture (g/100 g) 7.52 Ash (g/100 g) 1.37 Amino acid content (185 residues/1.000 residues)	-	Gel strength (g) 221.67 Viscosity (cP) 5.51	[52]

**Table 3:** Physico-chemical properties of microbial-origin biopolymers obtained from different agri-food waste and by-products.

Biopolymer	By-Product	Strain	Chemical Composition	Molecular Weight	Physical Properties	Ref.
bacterial cellulose	Lemon peels	*K. hansenii* *GA2016*	Moisture (% *w*/*w*) 7.25 Ash (% *w*/*w*) 7.23	-	Liquid-holding capacity (%(*w*/*w*)) in Water 886.00 Acetone 414.40 Dimethyl sulfoxide 904.40 Acetic acid 488.46 Average fiber diameter (nm) 59.98 Crystallinity (%) 88.55	[59]
	Mandarin peels	*K. hansenii* GA2016	Moisture (% *w*/*w*) 6.49 Ash (% *w*/*w*) 3.31	-	Liquid-holding capacity (%(*w*/*w*)) in Water 791.45 Acetone 307.80 Dimethyl sulfoxide 889.02 Acetic acid 611.01 Average fiber diameter (nm) 66.32 Crystallinity (%) 79.48	[59]
	Orange peels	*K. hansenii* *GA2016*	Moisture (% *w*/*w*) 7.73 Ash (% *w*/*w*) 9.01	-	Liquid-holding capacity (%(*w*/*w*)) in Water 595.76 Acetone 306.97 Dimethyl sulfoxide 574.18 Acetic acid 516.75 Average fiber diameter (nm) 47.92 Crystallinity (%) 91.54	[59]
	Grapefruit peels	*K. hansenii* *GA2016*	Moisture (% *w*/*w*) 8.06 Ash (% *w*/*w*) 4.82	-	Liquid-holding capacity (%(*w*/*w*)) in Water 705.17 Acetone 332.36 Dimethyl sulfoxide 792.84 Acetic acid 513.71 Average fiber diameter (nm) 55.45 Crystallinity (%) 91.96	[59]
Polyhydroxyalkanoates	Orange peels	recombinant *Escherichia coli* JM109	PHB	10 ÷ 3000 kDa	T_g_ = −6.8 ÷ 6.8 °C T_cc_ = 92 ÷ 99.3 °C T_m_ = 167 ÷ 172.6 °C χ = 12.3 ÷ 38.4%	[60]
	Red grape pomace	*Cupriavidus necator*	PHB		T_g_ = −3.1 ÷ 4.93 °C T_c_ = 91 ÷ 102.4 °C T_m_ = 164.5 ÷ 175.0 °C T_d_ = 279 ÷ 287 °C χ = 31.9 ÷ 46.5%	[61]
	Grape pomace and grape seeds	*Halomonas halophila, Halomonas organivorans, Cupriavidus necator*	PHB	252.4 ÷ 512.2 kDa		[62]
	Date waste	*Haloferax mediterranei*	PHBV (18 mol%., 3 HV)	746.0 kDa	T_g_ = −9 ÷ 17 °C T_m_ = 148.1 °C χ = 26.5% σ_b_ = 10.7 MPa ε_b_ = 1%	[63]
	Avocado seed waste	*Cobetia amphilecti*	3-HB, PHB	1831 kDa		[64]
	Apple pomace	*Pseudomonas putida*	mcl-PHA (3 HD = 60.90 mol%, 3 HO = 23.43 mol%, HDD = 6.94 mol%, HDD_(=)_ = 4.76 mol%, 3 HH = 3.19 mol%, HTD_(=)_ = 0.77 mol%)	4.7 × 10^4^ kDa	T_g_ = −42 °C T_m_ = 47 °C E’ = 360 MPa	[65]
	Apple pomace and potato peel liquor	*Cupriavidus necator*	PHBV		T_m_ = 143.4 °C T_d_ = 253 °C χ = 9.6%	[66]
	Banana peels	*Zobellellae tiwanensis*	PHB		T_m_ = 169 °C T_d_ = 200 °C χ = 34.38% E = 776.6 MPa σ_b_ = 10.3 MPa ε_b_ = 1.4%	[67]
	Fruit waste	*Paraburkholderia sacchari*	PHB	455.8 ÷ 484.4 kDa	T_m_ = 172.1 ÷ 175.6 °C T_c_ = 77.1 ÷ 88.5 °C T_cc_ = 92.2 ÷ 96.2 °C χ = 57.3 ÷ 60.6% E = 827.9 ÷ 1992.7 MPa σ_b_ = 13.3 ÷ 28.7 MPa ε_b_ = 3.6 ÷ 14.8%	[68]
	Cassava Peel Waste	*Cupriavidus necator*	PHB		T_g_ = −24.2 ÷ −19.3 °C T_m_ = 69.5 ÷ 109.3 °C	[69]

## Data Availability

Available data are presented in the manuscript.

## Abbreviations

The following abbreviations are used in this manuscript:

CWS	cactus fruit waste seed
CMF	cellulose microfibril
CNF	cellulose nanofibril
CNW	cellulose nanowhisker
DD	degree of deacetylation
DM	degree of methyl esterification
MCC	microcrystalline cellulose
MMC	mixed microbial consortium
M_w_	molecular weight
NCC	nanocrystalline cellulose
PHBV	poly(3-hydroxybutyrate-co-3-hydroxyvalerate)
PBAT	polybutylene adipate terephthalate
PBS	polybutylene succinate
PCL	polycaprolactone
PCL	polycaprolactone
PEA	polyester amide
PHA	polyhydroxyalkanoate
PHB	polyhydroxybutyrate
PLA	polylactic acid
PVA	poly(vinyl alcohol)
SEM	scanning electron microscope
SBR	sequencing batch reactor
VFA	volatile fatty acid
WVP	water vapor permeability
